# Conditioning cortisol in humans: design and pilot study of a randomized controlled trial

**DOI:** 10.1186/s40814-018-0382-5

**Published:** 2019-01-18

**Authors:** J. Tekampe, H. van Middendorp, F. C. G. J. Sweep, S. H. P. P. Roerink, A. R. M. M. Hermus, A. W. M. Evers

**Affiliations:** 1 Health, Medical and Neuropsychology Unit, Faculty of Social and Behavioural Sciences, Institute of Psychology, Leiden University, P.O. Box 9555, 2300 RB, Leiden, The Netherlands; 2 Department of Medical Psychology, Radboud university medical center, Nijmegen, The Netherlands; 3 Department of Laboratory Medicine, Radboud university medical center, Nijmegen, The Netherlands; 4 Division of Endocrinology, Department of Internal Medicine, Radboud university medical center, Nijmegen, The Netherlands; 5 Department of Psychiatry, Leiden University Medical Center, Leiden, The Netherlands

**Keywords:** Endocrine conditioning, Cortisol, Placebo effect, Stress, HPA axis, Associative learning

## Abstract

**Background:**

Conditioning of physiological responses can be achieved by repeatedly pairing a previously neutral conditioned stimulus with the administration of a pharmacologically salient unconditioned stimulus. This type of conditioning has been effective for specific immune and endocrine responses, but results with regard to conditioning of cortisol, a key stress-regulatory parameter, are currently unclear. This paper describes a pharmacological conditioning design, optimized for the examination of effects of cortisol conditioning under both basal conditions and in response to stress.

**Methods:**

A double-blind randomized controlled conditioning paradigm aimed at conditioning of cortisol is conducted in 48 healthy female volunteers. During the acquisition phase, a gustatory stimulus (conditioned stimulus) is paired with hydrocortisone (100 mg, capsulated, unconditioned stimulus) three times before being administered together with placebo during three evocation sessions. To investigate possible effects of cortisol conditioning in response to stress, participants are exposed to the Trier Social Stress Test during the third evocation session. Primary outcome measure of this study is the mean area under the curve of salivary cortisol during the first two evocation sessions. As secondary outcomes, self-reported affect and stress as well as alpha-amylase are investigated. A pilot study was conducted to ensure that this design is feasible to be used in a larger study.

**Discussion:**

This study design provides an innovative opportunity to examine the conditioning of cortisol under basal conditions and in response to stress. Also, the possible effect of cortisol conditioning on secondary outcomes of self-reported affect and alpha-amylase can be investigated. If cortisol could successfully be conditioned, this would be of conceptual relevance, showing that hypothalamic pituitary adrenal (HPA) axis regulation can be influenced by associative learning processes. Eventually, this could also have important clinical implications for understanding and treating stress-related disorders in which HPA axis dysregulation might play a role.

**Trial registration:**

Nederlands Trial Register, NTR4651. Registered on 29 July 2014

**Electronic supplementary material:**

The online version of this article (10.1186/s40814-018-0382-5) contains supplementary material, which is available to authorized users.

## Background

Conditioning of physiological responses can be achieved by repeatedly pairing a previously neutral stimulus (e.g., taste or odor; conditioned stimulus, CS) with the administration of a pharmacologically salient stimulus (e.g., an immunomodulatory agent; unconditioned stimulus, UCS). After repeated pairing of CS and UCS, administration of the CS alone or in combination with a placebo pill evokes a conditioned response (CR), which can be either mimicking or counter balancing the unconditioned response (UCR) elicited by the pharmacological stimulus. This type of conditioning is effective for conditioning immunosuppressive [1–4], allergic [5, 6], and glycemic responses [7–9]. The conditioned physiological responses observed in these studies are also called learned placebo effects, as the effective qualities of the pharmacological stimulus (UCS) are transferred to a previously inert stimulus (CS), which is often administered in combination with a placebo [10, 11]. Conceptually, they have important implications for the investigation of placebo effects, as they illustrate the close interaction between the central nervous system (CNS) in which the association between UCS and CS is thought to be taking place and peripheral functions regulating the UCR and, later, the CR [10]. In comparison to other placebo-inducing mechanisms, such as verbal suggestion, conditioning, and other learning mechanisms tend to induce larger placebo effects that can be evoked repeatedly [12]. Clinically, conditioned physiological responses may in time be used to reduce the amount of medication needed to ameliorate symptoms and thereby might also help to reduce unwanted side effects. First studies in this direction revealed promising results [13–18].

While conditioned immunosuppressive responses can reliably be replicated [1–4], results regarding the endocrine parameter cortisol have been less consistent [19–21], albeit not less relevant. Cortisol has been identified as an important target for conditioning, as there is a strong bi-directional communication between the CNS and the adrenal glands that produce and release cortisol via the hypothalamic pituitary adrenal (HPA) axis, which is seen as a prerequisite for conditioning. Clinically, dysregulation of the key stress-regulatory parameter cortisol is thought to be involved in a variety of stress-related disorders [22–24] and blunted cortisol responses to stressors are associated with the development and maintenance of autoimmune and chronic inflammatory diseases [25–27]. Also, interventions targeting cortisol have shown promising results including reduced negative affect in response to a short-term psychosocial stress task [28], reduced attentional bias toward angry faces in social phobia [29], and reduced frequency and intensity of intrusions in PTSD [30]. As the effects of interventions targeting cortisol emerge predominantly under stressful conditions, it would be of interest to investigate conditioning of cortisol not only under basal conditions, but also in response to stress. Successful conditioning of cortisol might thus not only provide new insights into the central regulation of the HPA axis, it might eventually also provide new ways to address dysregulation of the HPA axis in clinical settings, where it may become a valuable addition to existing treatment options for stress-related disorders.

Cortisol conditioning has been addressed by several animal studies, providing indications that the equivalent to cortisol in rodents, corticosterone, can be conditioned [31–33]. In humans, however, only very few studies have been conducted on this topic. One study has found conditioned cortisol decreases after conditioning with sumatriptan, which inhibits the release of cortisol [20]. However, another study, in which the glucocorticoid dexamethasone, which inhibits the release of cortisol, was used as UCS has led to inconclusive results, showing a statistically significant interaction effect across groups (conditioned vs. placebo control) and measurement (evocation vs. no evocation), but post hoc tests remained statistically non-significant [19]. A third study did not show significant increases in cortisol and noradrenaline after conditioning with corticotropin-releasing hormone, which stimulates the release of adrenocorticotropic hormone and in turn cortisol, although a post hoc analysis revealed that participants with above-median cortisol levels at baseline did show significantly increased cortisol production as a conditioned effect [21]. As the studies addressing conditioning of cortisol so far have all used different unconditioned stimuli, which affect cortisol release in different directions and possibly also via different regulatory mechanisms, it is difficult to compare the obtained results. In general, these studies provide indications that conditioning of cortisol might be possible, without allowing a clear conclusion at this time.

The current study design aims to build on previous research, using hydrocortisone as UCS, and examining conditioned responses not only under basal conditions, but also in response to psychosocial stress. Hydrocortisone was chosen as unconditioned stimulus as it is the pharmacological equivalent of cortisol. In the central nervous system, hydrocortisone binds to mineralocorticoid as well as glucocorticoid receptors, while dexamethasone, which was previously used to condition cortisol [19], binds to glucocorticoid receptors only. Also, previous studies using hydrocortisone to manipulate cortisol levels showed promising results with important implications for the treatment of stress-related disorders [28–30]. Including a psychosocial stress task in the design as a real-world challenge of the system in which the conditioned response is to occur, provides the opportunity to pre-test the possible clinical relevance of conditioning of the HPA axis in the future and to optimize the external validity of the study.

A double-blind, randomized, placebo-controlled study design is proposed, in which hydrocortisone (UCS) is repeatedly paired with a gustatory conditioned stimulus. As primary outcome, this study examines effects of conditioning on salivary cortisol under basal conditions. Secondary outcomes are cortisol in response to stress and self-reported affect, stress, and alpha-amylase both under basal conditions and in response to stress. Additionally, parameters indicating autonomic responses to stress (heart rate and skin conductance) are explored. The present paper describes the design of this study, including a small pilot study conducted to test its feasibility (see also [34]).

## Methods

The study protocol has been approved by the medical research ethics committee of the Leiden University Medical Center (LUMC; P14-020, NL47105.058.14). The study is conducted according to the principles of the Declaration of Helsinki (21.10.2008) and in accordance with the Dutch Medical Research Involving Human Subjects Act (WMO). The Standard Protocol Items: Recommendations for Interventional Trials 2013 (SPIRIT) Checklist is presented as Additional file 1. All data (e.g., questionnaires, laboratory results) are stored using anonymous participant identification codes and will be kept for a period of 15 years. The file linking the participant identification codes and personal data is managed by the researchers and the data manager, and is locked for access by others. In accordance with the Central Committee on Research Involving Human Subjects statement on publication policy, the results of this study will be submitted for publication in peer-reviewed journals, regardless of confirmation or disconfirmation of the hypotheses.

### Study participants

In view of the higher prevalence of stress-related disorders in women than men [35], this study is conducted in female participants. To be eligible, participants need to be between 18 and 30 years of age, premenopausal, and capable of understanding and producing Dutch fluently. Potential participants are excluded if they have psychiatric (Diagnostic and Statistical Manual of Mental Disorders-IV, DSM-IV) conditions, are diagnosed with a somatic disease that might interfere with the participant’s safety and/or the study protocol, use medication including oral contraceptives or any kind of drugs, have recently experienced stressful life events, are or intend to become pregnant at the time of participation or engage in breast feeding, or suffer from a known hypersensitivity or allergy to one of the ingredients of the hydrocortisone and placebo capsule or the gustatory conditioned stimulus used in the study. Eligibility of potential participants is assessed during a screening appointment.

### Study design

#### Experimental phases

**Fig. 1:**
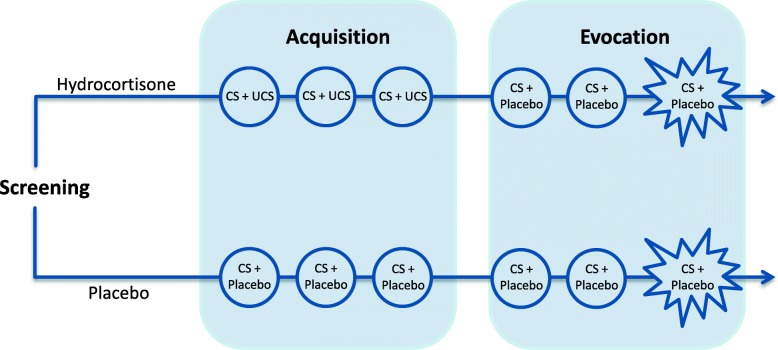
Overview of the study design. After screening, participants are randomized to either the hydrocortisone conditioning group or the placebo control group.  Each group undergoes six experimental sessions. During the acquisition phase (week 1), the hydrocortisone conditioning group is administered a capsule containing 100 mg of hydrocortisone (unconditioned stimulus, UCS) combined with a distinctively tasting beverage (conditioned stimulus, CS), whereas the placebo control group receives an identically looking placebo capsule and the same beverage. During the evocation phase (week 2), all participants receive a placebo capsule and the beverage.  In the final session, all participants are exposed to a psychosocial stress task

The design proposed for this study (see Fig. 1) is based on a widely used double-blind, randomized, placebo-controlled conditioning paradigm, consisting of two phases (e.g., [2, 4, 9]). The first phase is the acquisition phase (session 1 to 3), in which an association between the pharmacologically salient unconditioned stimulus (UCS) and the previously neutral conditioned stimulus (CS) is established by repeated paired administration. In the second phase, the evocation phase (session 4 to 6), it is tested whether administration of the CS with an identically-looking placebo pill elicits a conditioned response. In the current study, participants are additionally exposed to a psychosocial stress task during the final evocation session (session 6), in order to investigate whether conditioning affects the psychophysiological response to stress.

#### Conditioned stimulus (CS)

The CS used in this study is 150 ml of a distinctively tasting green beverage. The beverage consists of strawberry milk (Campina b.v., the Netherlands) to which yellow (chinolin yellow, Caelo, Germany) and blue (patent blue, Caelo, Germany) colorant, and lavender oil (KeyPharm Laboratories, Belgium) is added to achieve the green color and distinctive flavor. This beverage has previously been used as conditioned stimulus in several studies addressing conditioned immune responses [1–4, 15, 16, 36, 37].

#### Unconditioned stimulus (UCS)

The UCS used in this study is 100 mg of hydrocortisone. The dosage of 100 mg is chosen as it leads to a pronounced increase of cortisol without causing subjectively noticeable effects [38, 39]. Also, repeated administration of this dosage is unlikely to cause any unwanted side effects in healthy participants [40].

#### Randomization and blinding

This experiment is conducted in a double blind manner. Randomization and blinding as well as production of identically looking hydrocortisone and placebo pills is done by the Pharmacy of the Leiden University Medical Center. Pills are delivered in two containers, one for the acquisition and one for the evocation phase. Each container contains three pills, one for each day of the experiment. Containers are labeled with the participant and study code and the study phase in which the pills should be administered (week 1 for acquisition, week 2 for evocation). To check for blinding, at the end of the study, participants are asked whether they thought to have received hydrocortisone or placebo during each session of the experiment.

### Measurements and materials

#### Salivary cortisol and alpha-amylase

Cortisol and alpha-amylase are assessed under basal conditions and as markers of the psychophysiological response to stress by means of saliva samples [41, 42] collected with salivettes (Sarstedt, Rommelsdorf, Germany). Participants will be instructed to place the cotton swab contained in the salivette tube in their mouth and move it through their mouth using their tongue for 1 min. Participants are specifically instructed not to bite or chew on the cotton swab and not to touch it with their hands. Especially for stress-related research, the advantage of salivary measures compared to blood sampling is that the collection of saliva is non-invasive, inducing no or minimal amounts of stress [42]. Saliva samples are stored at − 80 °C at the Biobank in the LUMC.

#### Heart rate and skin conductance

Heart rate and skin conductance level are assessed as a measure of autonomic activity, using a non-invasive Biopac© apparatus consisting of the MP150 Data Acquisition System and the ECG100C Electrocardiogram Amplifier and the GSR100C module with a sampling rate of 1000 per second. For heart rate recordings, electrodes are applied to the participants’ body employing a Lead-II configuration (one on the chest, one on the ribs; no ground is needed because of the simultaneous recording of skin conductance) and a high-pass filter of 0.5 Hz is used. For skin conductance, two electrodes are being applied to the skin of the participants’ non-dominant hand. Gain is set to 5 μƱ/V, and a low-pass filter of 10 Hz is used.

#### Self-reported positive and negative affect

State positive and negative affect is assessed using the Dutch state version of the Positive and Negative Affect Schedule (PANAS, [43]). This validated and widely used self-report questionnaire asks for the level at which the participant experiences 10 negative emotion adjectives and 10 positive emotion adjectives at this moment, to be answered on a 5-point Likert scale (1 = “very slightly or not at all,” 5 = “extremely”) and takes about 2 min to complete.

#### Self-reported stress

Visual analog scales (VAS, [44]) are used to additionally measure stress-related items. The VAS were previously used by Childs et al. [45] and de Brouwer et al. [46] to assess seven psychological states at this moment (e.g., relaxed, nervous), anchored from “not at all,” reflecting a score of 0, to “very much so,” reflecting a score of 100.

#### Psychosocial stress task

The psychosocial stress task administered during the final evocation session (session 6) is the Trier Social Stress Test (TSST, [47]). Consisting of a 5-min preparation period, a 6-min prepared free speech, and a 4-min mental arithmetic task, the TSST has been shown to reliably elicit psychophysiological responses, including elevated cortisol and sympathetic activation [48, 49].

### Procedure

#### Recruitment and consent

Participants are recruited from the student population of Leiden University by means of printed and digital advertisements. When a person indicates interest in participating, she is provided with information about the study in writing and all appointments for the study are scheduled via telephone. During the first appointment, participants are informed about the study orally, invited to ask any additional questions, and asked to provide written informed consent to the experimenter. To rule out possible conscious expectancy effects of knowing about the conditioning procedure, all participants are told that the purpose of the experiment is to investigate the effects of repeated administration of hydrocortisone. No details about possible effects of hydrocortisone administration are given, except for the standardized information leaflet of hydrocortisone that is provided to participants as part of the standard informed consent procedure. All participants are informed that they can end their participation at any time during the study, without the need to provide a reason and without any negative consequences. Also, they are informed that the investigator could potentially decide to exclude them from further participation in the study for safety reasons. Might they occur, all adverse events are followed up until they have abated or until a stable situation has been reached. Depending on the event, follow-up may require referral to the general physician or a medical specialist. Possible damage to research subjects through injury or death caused by the study is covered by an insurance that is in accordance with the legal requirements in the Netherlands (Article 7 WMO and the Measure regarding Compulsory Insurance for Clinical Research in Humans of 23 June 2003).

#### Screening

Before entering the experiment, subjects who indicate interest to participate in the study are screened with questionnaires and a structured interview for the presence of any psychiatric (DSM-IV) condition [50] at the Faculty of Social and Behavioural Sciences of Leiden University. In addition, potential participants are screened for all somatic diseases that might interfere with the participant’s safety and/or the study protocol by a physician at the LUMC. In the 24 h before a study appointment, subjects are asked to refrain from drinking alcoholic beverages and using drugs. They are also instructed not to engage in physical exercise during the 12 h before an appointment. For the 2 h before an appointment, participants are asked not to eat heavy meals, drink caffeinated beverages, including tea, or to smoke cigarettes. During the screening, demographic characteristics of the participants and variables known to affect cortisol levels (such as menstrual cycle phase, BMI, smoking and perceived stress) are assessed using self-report questionnaires (e.g., Perceived Stress Scale, PSS, [51]). Subsequently, baseline measurements of self-reported affect and stress, saliva collection for measurement of cortisol and alpha-amylase and recordings of heart rate and skin conductance commence. If subjects are eligible to participate, they enter the randomization scheme.

#### Acquisition phase

Each of the three acquisition sessions follows the same regimen. Upon arrival at the laboratory at the Faculty of Social and Behavioural Sciences of Leiden University, the participant is asked to complete a self-report questionnaire measuring affect and seven VAS measuring stress. Then, a saliva sample for cortisol and alpha-amylase is taken. Next, participants are administered a pill containing either 100 mg of hydrocortisone or placebo paired with the gustatory CS. Subsequently, the participant is instructed to avoid potential activities that might interfere with conditioning for the next 4 h (e.g., drinking caffeinated or alcoholic beverages, consuming heavy meals, and exercising).

#### Evocation phase

**Fig. 2:**
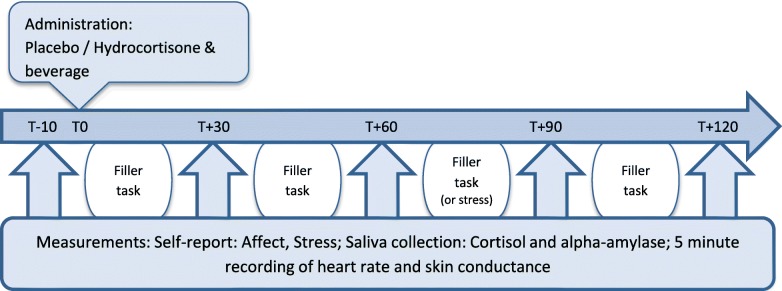
Measurement schedule for the evocation sessions: each of the evocation sessions has the same time schedule, with exception of the sixth session in which one filler task is replaced by the Trier Social Stress Test, and a sixth measurement is added at T + 150

A week after the acquisition phase, the three evocation sessions take place (on the same days of the week as the acquisition sessions). As shown in Fig. 2, each of the three evocation sessions follow the same regimen. First, participants are provided with electrodes to record heart rate and skin conductance throughout the session. Next, participants are asked to complete the self-report questionnaires on affect and stress again. Also, a saliva sample for cortisol and alpha-amylase measurement is taken and the participant is asked to sit still for a 5 min recording of heart rate and skin conductance (T-10). After this baseline measurement, participants are administered a placebo pill paired with the same distinctively tasting beverage as used in the acquisition sessions (T0). Self-report measurements of affect and stress, saliva collection, and heart rate and skin conductance recordings are repeated with intervals of 30 min (T + 30–T + 120). In evocation sessions 4 and 5, measurements are conducted 5 times, and in session 6 an extra measurement takes place 30 min after the fifth measurement. In the time between measurements, participants are asked to complete several undemanding and non-stressful filler tasks. In session 6, participants are additionally presented with the TSST.

#### Debriefing

After the last evocation session, participants are debriefed about the conditioning procedure. If participants choose to withdraw before completion of the study, they are debriefed as soon as their decision to withdraw is final. All participants will be informed about their group allocation and the study results when the study is finalized.

#### Statistical analysis

Main outcome parameter of this study is the area under the curve from ground (AUC_g_) of all salivary cortisol measurements in sessions 4 and 5, thus evocation under basal conditions. Data will be analyzed by an ANCOVA with condition (hydrocortisone or placebo) as independent variable and AUC_g_ of salivary cortisol as dependent variable. As the AUC_g_ of salivary cortisol from our pilot study showed a strong correlation with perceived stress (score on PSS during screening), PSS score and other potentially relevant covariates will be added to the analysis.

Secondary outcome parameters are AUC_g_ of salivary cortisol in session 6 and alpha-amylase, self-reported affect, and stress for which separate analyses will be conducted for measurements under basal conditions (sessions 4 and 5) and in response to stress (session 6). Additionally, effects of conditioning on the autonomic parameters heart rate and skin conductance will be explored. These analyses will be similar to those described for the primary outcome measure.

#### Sample size

In order to gain insight into the effect size of the group difference in basal cortisol after conditioning (primary outcome measures) that could be expected when conditioning with hydrocortisone, a pilot study was conducted using a similar design as in the current study. This pilot study in 10 female participants (7 receiving hydrocortisone in the acquisition phase and 3 receiving placebo) found a difference (Δm) in the AUC_g_ of cortisol during the first 2 evocation sessions of 6.14 with a weighted standard deviation of 11.68, resulting in an effect size of *d* = 0.53. Because of the preliminary character of this pilot study, findings of comparable studies on the effects of conditioning on endocrine outcomes (insulin, blood glucose; [7–9]) were also taken into account. In these comparable studies, effect sizes of *d* = 0.73 [9], *d* = 0.57 ([7]; insulin vs. placebo control group), and *d* = 0.77 [8] were found. Resultantly, we ran the power analysis with an averaged and conservatively rounded effect size of *d* = 0.60. Because a high correlation between the outcome variable AUC_g_ of cortisol under basal conditions and the subjectively perceived stress level (PSS) at baseline (*r* = .85) was found in our pilot study, a conservative estimate of this correlation (*r* = .70) was added as a design factor in the sample size calculation [52]. Based on these estimations, a sample size calculation for an independent samples *t* test indicated that with an alpha level of 0.05 and a power of 0.80, 46 participants would be needed. Due to block randomization, the total number of participants to be included was adjusted to 48.

#### Pilot study

A pilot study was conducted in 10 healthy female volunteers at the Radboud university medical center (Radboudumc) to gain information about the feasibility of the design, next to providing input for the potential effect size of the conditioned effect as described in the previous paragraph. The pilot study was ethically approved by the medical research ethics committee of the Radboudumc (2012/290, NL40509.091.12). Because basal cortisol (e.g., [53, 54]) and cortisol responses to the TSST without conditioning have already been investigated in previous research (e.g., [48, 49]), an unequal group allocation was chosen, with more participants being allocated to the hydrocortisone conditioning group (*n* = 7) than to the placebo control group (*n* = 3). Regarding the usefulness and practicality of the measurement schedule used in this study, the absence of missing values in any of the physiological assessments (cortisol, alpha-amylase, heart rate, and skin conductance) or self-report affect questionnaire (PANAS) indicates that the number and frequency of measurement points in this study was feasible. The check for blinding conducted at the end of the study indicated that none of the participants was able to guess reliably whether she had received hydrocortisone or placebo at any point of the study. None of the participants reported physical symptoms that could be attributed to the hydrocortisone treatment during the study.

While the design of the pilot study was the same as the design of the current study, some adaptations have been made in the study procedure. In the pilot study, the procedure of the acquisition sessions (session 1 through 3) was identical to the evocation sessions (sessions 4 and 5) as depicted in Fig. 2. Thus, each session comprised 5 measurement points, 1 every 30 min, and lasted approximately 2.5 h. The pilot data showed that administration of 100 mg hydrocortisone in the hydrocortisone conditioning group induced mean increases of 1237.66 mmol/l (sd = 485.45) from baseline in salivary cortisol within 120 min (measurements taken every 30 min), while none of the participants reported any subjectively noticeable (side-)effects of the hydrocortisone treatment. No changes in salivary cortisol were indicated in the placebo control group (mean difference from baseline − 2.28 mmol/l; sd = 1.12). Also, no changes in self-reported affect (PANAS) and alpha-amylase were indicated in both groups during the acquisition phase. Therefore, the acquisition sessions of the current study were limited to a baseline measurement of salivary cortisol and the standardized administration of CS and UCS, in order to significantly reduce participant burden.

## Discussion

To investigate conditioned responses of the hypothalamic pituitary adrenal (HPA) axis parameter cortisol, both under basal conditions and in response to psychosocial stress, a double-blind, randomized study design is proposed. The current study design aims to build on previous research, by investigating possible conditioned responses under basal conditions as well as in response to psychosocial stress, providing the opportunity to pre-test the possible clinical relevance of conditioning of the HPA axis in the future and to optimize the external validity of the study. Therefore, effects of conditioning on alpha-amylase, self-reported affect, and stress are assessed as well.

The design used in this study is based on previous studies in which immune and glycemic responses were classically conditioned (e.g., [2, 4, 9]). The conditioned stimulus (CS) used in this study is a distinctively tasting beverage also used in previous conditioning studies [1–4, 15, 16, 36, 37], providing a gustatory stimulus that is unfamiliar to participants and has therefore not been associated with other stimuli prior to this experiment. Unlike in previous studies on the conditioning of cortisol, hydrocortisone was chosen as the unconditioned stimulus (UCS) in this study. In our pilot study, administration of hydrocortisone during the acquisition phase led to a marked increase in salivary cortisol, while blinding of the participants and experimenters was not compromised, supporting its potential usefulness as UCS for conditioning cortisol. The number of acquisition and evocation sessions was also based on previous studies. Studies in humans have used between one [5, 6] and six [8] repetitions of paired administrations of the CS and UCS. Classical conditioning theory and preliminary data suggest that more paired administrations of CS and UCS during acquisition more reliably lead to conditioned responses [5]. This finding is reflected in the choice for three paired administrations of CS and UCS in this study, using multiple acquisitions while ensuring practical feasibility of the study and minimizing the burden for participants. In previous studies, time laps between the paired administrations varied between several minutes [8] to 1 or 2 weeks [19]. In the current study, considering the 8-h half-life of hydrocortisone, paired administrations of CS and UCS could only take place once per day in order to prevent a build-up of hydrocortisone in the participants. To keep paired administrations together as closely as possible, the acquisition sessions in this experiment take place on three consecutive days. After the acquisition phase and before the evocation phase, there is a drug washout period of 4 days to ensure measurements in the evocation phase are not influenced by residues of hydrocortisone or suppression of the HPA axis that might result from hydrocortisone treatment. As previous studies have shown that not all conditioned effects are evident after the first evocation [36], this study design includes three evocation sessions. To optimize conditioned effects in case stimuli other than the beverage have also become associated with the UCS, the procedure surrounding the administration of CS and UCS during these sessions as well as the lab environment and the experimenter are the same during acquisition and evocation sessions for each participant. This highly repetitive nature of the study procedures has the additional advantage that all events occurring during the study appointments (with exception of the TSST in the last session) are highly predictable for the participants, and thereby minimizes effects of novelty or anticipation. To investigate conditioned responses in reaction to stress, participants are exposed to the Trier Social Stress Test (TSST) during the last evocation session. Cortisol is a key-stress regulatory parameter and its dysregulation is thought to be involved in a variety of stress-related disorders [22–24]. Therefore, in this study, conditioned responses are investigated not only under basal conditions but also in response to psychosocial stress which provides the opportunity to pre-test the possible clinical relevance of conditioning of the HPA axis in the future and to optimize the external validity of the study. Regarding the feasibility of the overall design used in this study, the results of the pilot study have demonstrated that this design is feasible to be used in a larger study. No participants reported side effects of hydrocortisone treatment and only three participants dropped out, all with reasons unrelated to the study protocol. Finally, a power analysis based on findings from the pilot study as well as previous studies indicated a number of 48 participants to be included in this study, making this the largest study on conditioning of the HPA axis conducted thus far.

This study includes only healthy female volunteers with a limited age range. This benefits the homogeneity of the sample and therefore increases chances of finding reliable effects. Also, women show a higher prevalence of stress-related disorders than men [35] and are, therefore, a more relevant group to target for this study. Menstrual cycle phase, a factor known to affect cortisol responses to stress, is noted, but the experimental sessions are not scheduled in a specific phase of the menstrual cycle. Furthermore, all measurements in this study are non-invasive. More invasive procedures such as blood sampling is known to cause stress in most individuals and could thereby possibly trigger responses of the HPA axis [53], which might confound the results and possibly disrupt the conditioning process.

If cortisol could successfully be conditioned, this would be of conceptual relevance, showing that hypothalamic pituitary adrenal axis regulation can be influenced by associative learning processes. It would also provide opportunities for future research and clinical applications. As cortisol is a key stress-regulatory parameter and HPA axis dysregulation might play a role in stress-related disorders, cortisol conditioning could be investigated experimentally in patient populations, possibly using a stress-inducing challenge relevant and appropriate for the specific group (e.g., exposure to a phobic stimulus). Not only could this provide further insight into mechanisms underlying stress-related disorders, it may also identify cortisol conditioning as a valuable addition to existing cognitive-behavioral treatments of stress-related disorders.

To conclude, the proposed study design aims to provide more clarity on the possibility to condition HPA axis responses in humans and expands previous studies by investigating possible conditioned responses under basal conditions as well as in response to psychosocial stress. Results from our pilot study have already demonstrated that this design is feasible, providing sufficient ground to examine this design further in a larger randomized controlled trial (see also [34]). If cortisol could successfully be conditioned, this would be of conceptual relevance, showing that HPA axis regulation can be influenced by associative learning processes. Eventually, this could also have important clinical implications for understanding and treating stress-related disorders in which HPA axis dysregulation might play a role.

## Additional file

Additional file 1: SPIRIT 2013 checklist: recommended items to address in a clinical trial protocol and related documents. (DOC 121 kb)
